# The impact on health of employment and welfare transitions for those receiving out-of-work disability benefits in the UK

**DOI:** 10.1016/j.socscimed.2016.05.042

**Published:** 2016-08

**Authors:** Esther Curnock, Alastair H. Leyland, Frank Popham

**Affiliations:** MRC/CSO Social and Public Health Sciences Unit, University of Glasgow, UK

**Keywords:** Employment, Unemployment, Disability/disabled persons, Social welfare/social security, Health status, Labour market status, United Kingdom, Difference-in-difference

## Abstract

Employment status has a dynamic relationship with health and disability. There has been a striking increase in the working age population receiving out-of-work disability benefits in many countries, including the UK. In response, recent UK welfare reforms have tightened eligibility criteria and introduced new conditions for benefit receipt linked to participation in return-to-work activities. Positive and negative impacts have been suggested but there is a lack of high quality evidence of the health impact when those receiving disability benefits move towards labour market participation. Using four waves of the UK’s Understanding Society panel survey (2009–2013) three different types of employment and welfare transition were analysed in order to identify their impact on health. A difference-in-difference approach was used to compare change between treatment and control groups in mental and physical health using the SF-12. To strengthen causal inference, sensitivity checks for common trends used pre-baseline data and propensity score matching. Transitions from disability benefits to employment (n = 124) were associated on average with an improvement in the SF12 mental health score of 5.94 points (95% CI = 3.52–8.36), and an improvement in the physical health score of 2.83 points (95% CI = 0.85–4.81) compared with those remaining on disability benefits (n = 1545). Transitions to unemployed status (n = 153) were associated with a significant improvement in mental health (3.14, 95% CI = 1.17–5.11) but not physical health. No health differences were detected for those who moved on to the new out-of-work disability benefit. It remains rare for disability benefit recipients to return to the labour market, but our results indicate that for those that do, such transitions may improve health, particularly mental health. Understanding the mechanisms behind this relationship will be important for informing policies to ensure both work and welfare are ‘good for health’ for this group.

## Highlights

• High working-age disability benefit rates affect many welfare state countries.
• Employment transitions from disability benefit receipt are under-researched.
• We analysed three types of labour market transition using difference-in-difference.
• Transitions to employment improved health, particularly mental health.

## 1 Introduction

Increasing employment levels and reducing social security spending are policy priorities within many OECD economies. Employment participation is seen as a route out of poverty and social exclusion, as well as an obligation of citizenship (Grover and Piggott, 2013, Newman, 2011). Within public health and policy circles there has been a heated debate sparked by the question ‘is work good for health and wellbeing?’ (Waddell and Burton, 2006). Some commentators argue that negative aspects of work have been ignored, particularly when the *‘work is good for health’* mantra has been extended to the population with chronic illness and disabilities (Grover and Piggott, 2013), but there is little direct research in this area. Within some European countries up to 12% of the working age population receive disability benefit payments, and welfare states globally often spend more on sickness and disability benefits than on unemployment benefits (OECD, 2010). In response many OECD countries have introduced reforms to disability benefits, characterised by payments attached to mandatory participation in employability activities, a shift to assessing work capacity rather than disability, and more frequent reassessment (OECD, 2010). The UK is a pertinent example of this policy trend with the introduction of Employment and Support Allowance (ESA) replacing Incapacity Benefit (IB). From a public health perspective disability benefit welfare reforms affect a section of the working-age population implicitly comprised of those with the greatest health disadvantages. In Great Britain over 2.5 million people (6.3% of the working age population) receive these out-of-work disability benefits (DWP, 2015). Given the magnitude of the disability benefit welfare reforms it is of critical importance to increase our understanding of the health impacts on those affected. We study transitions into employment or into unemployment as well as transitions from IB to the replacement benefit ESA.

### 1.1 Reforms to disability benefit in the UK

Employment and Support Allowance (ESA) is the current income-replacement benefit paid to working-age people in the UK who are out-of-work because of long-term ill health or disability. ESA replaced Incapacity Benefit (IB) and was introduced for new applicants in 2008 (herein ESA and IB will be referred to collectively as ‘disability benefits’). Since 2011 those already receiving IB have been reassessed to decide whether they are eligible for ESA through a process known as the Work Capability Assessment (WCA). If the WCA finds an individual ‘fit for work’ they are not eligible for ESA and they will usually apply for unemployment benefit unless they can find employment (Barr et al., 2015a, Barr et al., 2015b). Those eligible for ESA are allocated into either the ‘Work Related Activity Group’ if they are judged to have limited capability for work; or the ‘Support Group’ if they have limited capability for ‘work-related activity’. Eligibility for out-of-work disability benefits tightened with the introduction of ESA and 64% of new applicants were found fit-for-work when ESA was first introduced (Work and Pensions Committee, 2014). Many of these decisions were contested and overturned by appeal tribunals. The WCA underwent several changes following a number of independent reviews and by 2013 the proportion of new applicants found fit-for-work had reduced to 27% (Work and Pensions Committee, 2014). For those receiving ESA allocated to the Work Related Activity Group, payments are attached to conditions (participation in work-focussed interviews and activities), with financial penalties if they fail to comply. Until 2012, no time limit was imposed for receipt of ESA, however, since then a one year limit has been imposed for those receiving the benefit on the basis of their individual national insurance contributions whereas those receiving ESA on the basis of means-testing continue without a time limit. Paid work is permitted whilst receiving ESA if an individual works less than 16 h and earns up to £107 per week. Permitted work can continue indefinitely for those in the support group, but otherwise is usually allowed for up to 52 weeks. See (Kennedy, 2014) for further background on the UK reforms.

### 1.2 The health impact of employment transitions

The implementation of the reforms is relatively recent and is ongoing; as such there is a lack of quantitative research regarding their health impact. Emerging qualitative work suggests the UK disability welfare reforms risk worsening wellbeing and increasing the stigma attached to benefit receipt (Garthwaite, 2014, Garthwaite, 2015, Patrick, 2011). A substantial body of research debates the individual and structural determinants of returns to labour market participation for the long-term sick and disabled (Bambra, 2011, Barr et al., 2010, Beatty et al., 2000, Kemp and Davidson, 2010, Lindsay et al., 2015), but this literature does not look directly at the impact on health of labour market participation.

Unemployment and transitions into worklessness are known to be associated with increased morbidity and mortality, worse self-rated health, as well as reduced social activity and social support (Bambra, 2011, Bartley, 1994, Booker and Sacker, 2012, Clemens et al., 2015, Popham and Bambra, 2010, Popham et al., 2012, Roelfs et al., 2011, Schuring et al., 2015, van Rijn et al., 2014). This body of evidence is well-established and includes systematic reviews and robust meta-analyses, although the direction of causality may still be contested. Drawing on evidence of the health effects of unemployment, policymakers may assume that the reverse relationship holds, and advocate re-employment as a means to improve health (van der Noordt et al., 2014, Virtanen, 2014, Vuori et al., 2015), however, there is less direct evidence on the health effects of employment, and research findings are more mixed. Longitudinal studies have shown transitions from unemployment into employment are associated with improved psychological wellbeing (Flint et al., 2013, Thomas et al., 2005), mental health (Schuring et al., 2011, van der Noordt et al., 2014), quality of life (Carlier et al., 2013, Flint et al., 2013), life satisfaction (Gebel and Voßemer, 2014, Grün et al., 2010), reduced depression and other mental health symptoms (Huber et al., 2011, Rueda et al., 2012). The evidence base for positive physical health impacts of unemployment to employment transitions is weaker (Carlier et al., 2013, Rueda et al., 2012, Schuring et al., 2011). Studies have shown a positive impact on self-rated health (Carlier et al., 2013, Ki et al., 2012, Rueda et al., 2012, Schuring et al., 2011, Schuring et al., 2015) as a proxy measure of physical health. A recent systematic review concluded there was strong evidence for the positive effects of transitions from unemployment to employment on mental health, but the evidence for employment having a positive impact on physical health and general health was insufficient (van der Noordt et al., 2014).

Transitions into employment may have greater health benefits when baseline health is worse (Huber et al., 2011, Milner et al., 2014, Schuring et al., 2011), but the evidence summarised thus far has focussed on transitions between unemployment and employment; fewer quantitative studies have specifically looked at the effects of transitions into employment for those economically inactive due to long-term sickness and disability. A positive health impact is associated with transitions from ‘inactivity’ into employment (Ki et al., 2012, Milner et al., 2014). However, this group is very heterogeneous making it difficult to draw meaningful conclusions regarding the impact for disability benefit recipients, as the inactive encompass not just those with long term sickness and disability but also people looking after home and family, students and those who retire early. An exception to this in the UK is the study by Flint et al. which used British Household Panel Survey data and found moving back into employment from an economically inactive category of ‘permanent sickness’ was associated with improved mental wellbeing as measured by the GHQ-12 (Flint et al., 2013). Research investigating the health impact of transitions from long-term sickness and disability into unemployment is equally sparse. In many countries there are financial and bureaucratic disincentives to make this transition (OECD, 2010). Ki et al. found that transitions from inactivity (again comprising a heterogeneous population) into unemployment were associated with worse health for women, whilst the effect on men was not statistically significant (Ki et al., 2012). Transitions from inactivity into unemployment have also been associated with worse psychological wellbeing (Booker and Sacker, 2012). In contrast, Flint et al. found that moving from inactivity due to permanent sickness into unemployment was not associated with any significant change in psychological wellbeing (Flint et al., 2013).

None of the longitudinal literature reviewed looked specifically at the health outcomes of labour market transitions for the group targeted by benefit reforms – those receiving out-of-work disability benefits. Return-to-work intervention trials involving those receiving disability benefits have focussed on employment outcomes rather than health outcomes, and evaluations of ‘health first’ interventions have assessed health measures before and after programme participation, but not health outcomes associated with employment transitions themselves (Bambra et al., 2005, NICE., 2009, Purdie and Kellett, 2015, Warren et al., 2014). One recent paper found regions with higher rates of IB reassessment had poorer mental health outcomes (Barr et al., 2015a, Barr et al., 2015b), but clear causal relationships cannot be drawn as the study was based on regional trends.

The lack of evidence regarding the health impacts of transitions from disability benefits into either unemployment or employment is significant given the policy emphasis on shifting this group towards labour market activity. Three fundamental problems lie behind the evidence gap. First, there is a reciprocal two-way relationship between economic status and health which makes questions of causality difficult to disentangle - health is an important determinant of transitions both into and out of all categories of economic status (Beatty and Fothergill, 2011, Gebel and Voßemer, 2014, Kemp and Davidson, 2010, Ki et al., 2011, Schuring et al., 2011, Thomas et al., 2005, van der Noordt et al., 2014, van Rijn et al., 2014). Second, longitudinal panel studies are often limited by the small numbers making such transitions within their sample (Milner et al., 2014, Thomas et al., 2005, Virtanen, 2014). Studies looking at the health impact of unemployment to employment transitions may specifically exclude those who are categorised as long-term sick and disabled (Gebel and Voßemer, 2014, Huber et al., 2011, Schuring et al., 2011). Third, in many countries there are limited data sources that combine both good quality health and welfare information. In the UK routine administrative health records do not include sufficient data on welfare receipt and employment status; routine UK health surveys do not include adequate benefit data (for example, they do not distinguish out-of-work disability benefits from benefits that may be received whilst in work); and, as yet, data linkage has not taken place between UK government administrative datasets and health records.

In summary, research evidence suggests that unemployment has a significant negative impact on health, while returning to employment is beneficial to psychological health. However, there is little robust evidence regarding the health impact of transitions to employment or unemployment from receiving disability benefits or between different disability benefits. The recently collected panel data of the Understanding Society survey contain specific indicators of disability benefit status and detailed health outcome data and has a sufficient combined sample size to analyse rare transitions from disability benefits, providing an opportunity for analyses which other data sources have not yet permitted and which have relevance to contexts where reforms seek to shift the relationship between disability, work and welfare.

### 1.3 Aim and hypotheses

The aim of this research was to look at the health impact of transitions towards labour market participation by conducting three different analyses for the group that have been targeted by the UK disability benefits welfare reforms:

Analysis 1: Transition from receiving out-of-work disability benefits (IB or ESA) to employment.

Analysis 2: Transition from receiving out-of-work disability benefits (IB or ESA) to unemployment.

Analysis 3: Transition from receiving IB to ESA.

Based on the existing literature, the following hypotheses were formed:

i. *On average, transitions to employment from receiving disability benefits are beneficial for health, compared to remaining on disability benefits (analysis 1).*

The research literature suggests there are health benefits associated with the transition into employment from unemployment and points towards a similar positive health impact for transitions from inactivity, particularly for mental health. The mechanisms underlying this relationship are likely to apply to those receiving disability benefits.

ii. *On average, transitions to unemployment from receiving disability benefits will have no impact on health outcomes, compared to remaining on disability benefits (analysis 2)*

The research literature suggests worse health may be associated with transitions from inactivity into unemployment, but there is no clear evidence regarding those receiving disability benefits. Unemployment welfare benefits in the UK are more insecure and of lower financial value. However, this group will include those with improving health that are found fit for work and (notwithstanding the study design) this may counteract potential negative impacts.

iii. *On average, the impact on health of transitions from IB to ESA will be small and not differ from those remaining on IB (analysis 3)*

The transition from IB to ESA may have both potential negative and positive influences on health. It is difficult to make prior predictions on the direction of this effect, but any difference between the groups is likely to be small.

## 2 Methods

### 2.1 Study design

The study design sought to minimise risks of bias, confounding and random error and to enhance a causal interpretation of exposure and outcome associations. Within a counterfactual framework, use of a difference-in-difference (DiD) design compares changes over time (t0 to t1) between exposed (treatment, d1) and unexposed (control, d0) groups who could have received the treatment but did not. The use of ‘before & after’ data collected from individuals removes fixed individual differences, including those that are unobserved. The design also removes time effects that impact both treatment and control groups in the same way. Drawing causal inferences from a DiD design relies on the assumption that control and treatment groups would behave in the same way if the treatment exposure did not take place. Two additional sensitivity analyses checked this common trends assumption. First, inspecting the trend of the outcome measures for the treatment and control groups prior to the intervention period (‘pre-baseline’) in a subsample where data were available, and second, using propensity score matching to adjust for baseline differences between the control and treatment groups. The study design was applied separately to each of the three analyses.

### 2.2 Sample

Data were drawn from four waves of the UK panel survey ‘Understanding Society’ (Institute for Social and Economic Research and NatCen Social Research, 2014), an annual longitudinal panel survey of people living in households in the UK, designed to be nationally representative through a stratified, clustered, equal probability sample (Buck and McFall 2011). Data collection in each of the waves takes place over two years: wave 1 2009–2010 (n = 50,994); wave 2 2010–2011 (n = 54,597); wave 3 2011–2012 (n = 49,739) and wave 4 2012–2013 (n = 47,157). To enable an adequate sample size, transition data across the four waves of the longitudinal datasets were pooled by matching individual adult (≥16 years) respondent datasets from each wave on the unique person identifier (web Appendix 1). The scale of the survey provides a sufficient sample size for the relatively small proportion who had complete exposure and outcome data at two consecutives waves and who make the transitions of interest in each analysis (n > 100 in the treatment groups, n > 1000 in the control groups; see results for detail). Individuals aged above retirement age at baseline (>59 years for females, and >64 years for males) were excluded. Exposure categories of employed, unemployed, and IB and ESA receipt were constructed based on self-reported survey responses (further detail in web Appendix 2). Outcome measures were based on the ‘Short Form-12’ (SF-12) self-completed questionnaire, with individual responses converted to physical and mental health summary scores on a scale from 0 (low functioning) to 100 (high functioning), with a mean population score of 50 (Jenkinson et al., 1997). Of those with exposure data allocated into a control or treatment group, the proportion missing health outcome data ranged from 23% to 33% (web Appendix 3). Baseline (t0) SF-12 scores for those with and without follow-up (t1) scores were checked for each analysis, as were follow-up scores for those with and without baseline scores (web Appendix 4).

### 2.3 Analyses

For each of the three analyses, difference in change in SF-12 physical and mental summary scores between baseline (t0) and one year follow-up (t1) between the control group and treatment group were calculated. For the treatment ‘becoming employed’ (IB/ESA at t0 and employed at t1), and the treatment ‘becoming unemployed’ (IB/ESA at t0 and unemployed at t1), comparisons were with a control group comprised of individuals who were receiving IB/ESA at both t0 and t1. For the treatment ‘moving to ESA’ (IB at t0 and ESA at t1), comparisons were made with a control group comprised of individuals who were receiving IB at both t0 and t1. The difference-in-difference estimate (DiD) can be derived by simple arithmetic from the mean health scores for the treatment and control group at t0 and t1. Standard errors and confidence intervals were calculated using regressions on the change score with clustering on the individual identifier. As a sensitivity test to strengthen the common trends assumption a subsample that had pre-baseline health data a year before (i.e. those with three consecutive waves of data providing t–1, as well as t0 and t1 health scores) was used to inspect the assumption of a common trend prior to t0. This was confirmed by estimating the DiD for the pre-treatment period (applying a placebo-treatment to t–1 to t0). Finally, propensity score matching was used to adjust for baseline differences at t0 between groups as an extension of the difference-in-difference analysis. The aim was to include all available observable characteristics acting as potential confounders of the relationship between employment transition and change in health (factors with an association both to treatment exposure and the outcome). The covariates included in the final iteration of the propensity score were sex, age, education, marital status, UK birth status, housing tenure (whether a house owner or not), number of own children in the household, gross monthly individual income, social class, years since last job, region of residence, longstanding illness or disability, psychological wellbeing (GHQ score 0–36), and total disability count (web Appendix 2 for detail on covariates). Amongst those who had complete exposure and health outcome data in the main sample, 10% had missing covariate data and were excluded from the propensity score sensitivity analysis (web Appendix 3 & 5).

Propensity scores were estimated for each individual and checked for the balance of the distribution of scores between the control and treatment groups, as well tests of the matching strategy quality, using the Stata user-written programmes ‘pscore’, ‘psmatch2’ and ‘pstest’ (Becker and Ichino, 2002, Garrido et al., 2014, Sianesi and Leuven, 2003). Iterative modifications were made (blind to outcome estimates) until the strategy resulting in the lowest balance of bias and variance between groups was identified (for detail web Appendices 6–10). The psmatch2 programme generates the propensity score matched difference-in-difference estimate (DiD-PSM), but to enable the calculation of clustered standard errors regressions were run on the health outcome change score with the matched propensity score weight (generated by psmatch2) applied as a probability weight. Alternative methods calculating bias-corrected standard errors (using bootstrapping) and robust ‘Abadie-Imbens’ (AI) standard errors (using the Stata ‘teffects psmatch’ programme) were also checked (web Appendix 11). This process was repeated for mental and physical health outcomes separately for each of the three analyses. All analyses were undertaken using Stata version 13 (StataCorp, 2013).

## 3 Results

Those who transitioned to employment had better physical and mental SF-12 health scores at baseline than those remaining on disability benefits. This was greatest for physical health where there was a ten point difference between treatment and control groups at baseline (p < 0.01). For those that transitioned to unemployment the mean baseline mental health score was similar to that of those who remained on disability benefits (p = 0.67), but their physical health score was substantially better than the control group (p < 0.01), and was only slightly lower than the group that transitioned to employment. The group that transitioned from IB to ESA had slightly worse mental health (p = 0.02) at baseline than the group that remained on IB (Table 1).

**Fig. 1:**
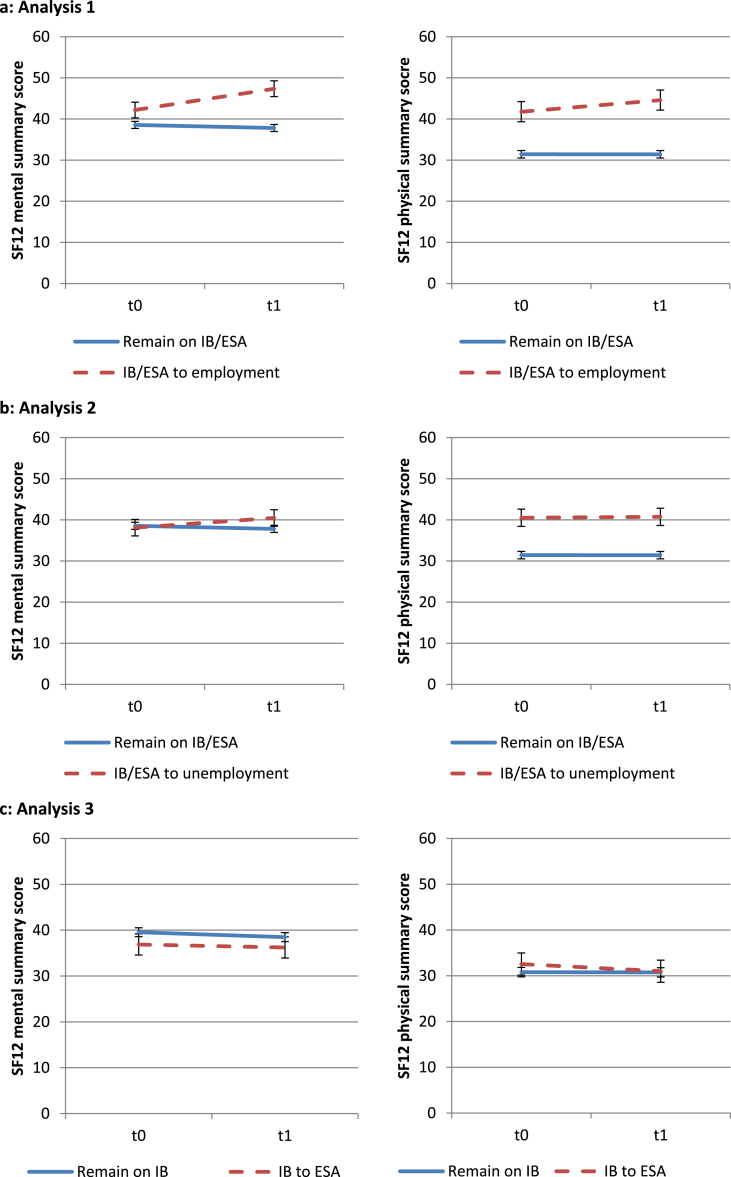
a–c: SF12 mental and physical health scores at baseline (t0) and follow-up (t1) for analyses 1–3 (error bars show 95% confidence intervals; IB = Incapacity Benefit, ESA = Employment & Support Allowance).

Transitions from disability benefits to employment (analysis 1) were associated on average with an improvement in the SF-12 mental and physical health scores compared with those who remained on disability benefits (Table 1, Fig. 1a). Transitions to unemployed status (analysis 2) were associated on average with an improvement in mental health but no significant difference in physical health (Table 1, Fig. 1b). No statistically significant differences in mental or physical health were associated on average with transitions from IB to ESA (analysis 3) compared with those who remained on IB (Table 1, Fig. 1c).

### 3.1 Sensitivity checks

**Fig. 2:**
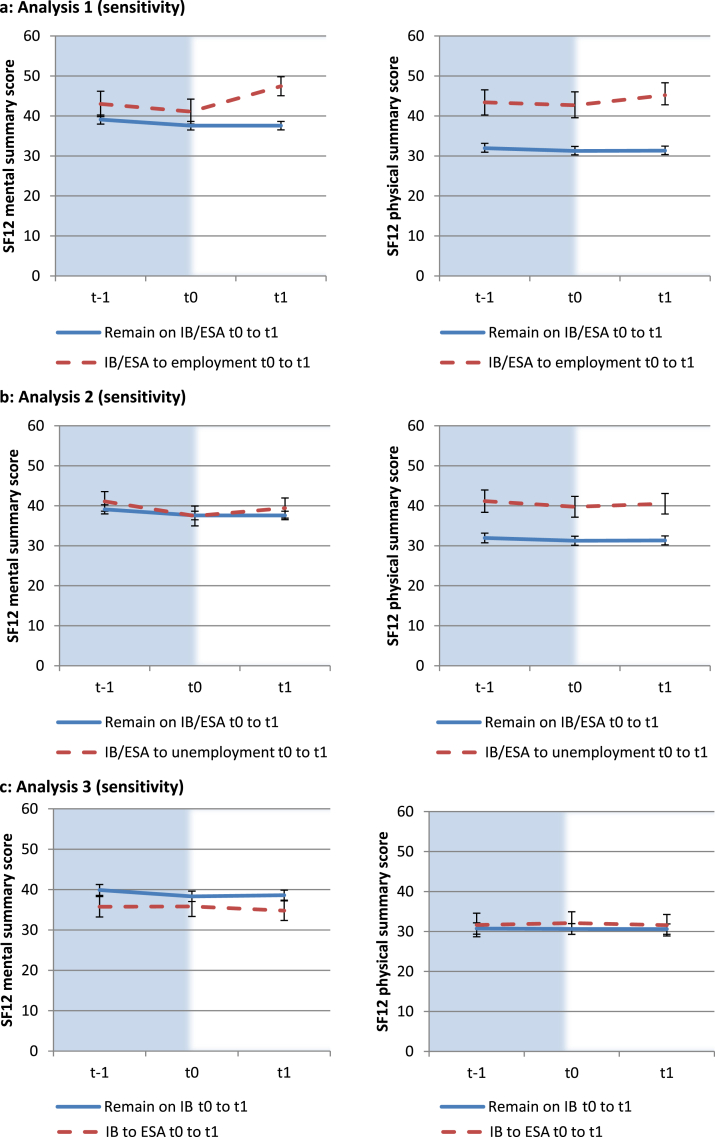
a–c: SF12 mental and physical health scores at pre-baseline (t–1), baseline (t0) and follow-up (t1) for analyses 1–3.

Visual inspection of the average health score plots of the treatment and control groups for each analysis at t–1, t0 and t1 (for the subsample that had pre-baseline data) suggests the common trends assumption is valid (Fig. 2a–c). In particular, although those who transitioned to employment had a higher baseline mental and physical health score than the control group, prior to the employment transition they were on a similar trajectory of slight decline (analysis 1). The visual impression is confirmed when a placebo treatment is applied to the pre-intervention period (t–1 to t0) where no statistically significant DiD estimate is found (web Appendix 12).

Compared with the control group, prior to matching, those who transitioned from disability benefits to employment were on average younger, better educated, less deprived, had more children in the household, and were healthier. They were also more recently connected to the labour market than the control group, with many indicating some form of paid employment at the first wave of survey participation. Those transitioning from disability benefits to unemployment were also on average younger and healthier than the control group, but were more poorly educated, were more deprived and less likely to be married. In analysis 3 the characteristics of the treatment and control groups were more similar prior to matching. Summary statistics of baseline covariates for the treatment and control groups in all analyses can be found in web Appendix 13. The propensity score matching strategy sought to reduce the bias between groups for each covariate. A kernel weighting with a 0.03 bandwidth emerged as the optimal approach when the alternative matching strategies were tested for each analysis. When the kernel weighting was applied as a matching strategy, ten individuals in the treatment group in analysis 1 were excluded as they were off common-support, i.e. no equivalent controls could be found for these individuals. This group were younger, more affluent, and had better health indicators at baseline than the rest of the treatment group. Once the propensity score matching strategy was finalised the difference-in-difference estimates were re-calculated (DiD-PSM); the statistically significant transitions were unchanged from the main DiD analyses although each of the estimates reduced slightly (Table 1). Alternative methods for calculating the standard error did not alter the findings (web Appendix 11). The propensity score strategy was also applied to analysis 1 for the sensitivity subsample group, using pre-baseline (t–1) data to construct the propensity score (web Appendices 14 and 15). For this subsample the DiD-PSM estimate for mental health remained statistically significant (web Appendix 16).

## 4 Discussion

### 4.1 Summary

This study sought to identify the impacts of transitions towards increased labour market participation on the health of the population who were receiving out-of-work disability welfare benefits in a period of major welfare reform. Using four waves of the Understanding Society survey (2009–2013), three different types of transition were analysed using difference-in-difference methods. Sensitivity analyses extended this approach with checks for common trends by examining pre-baseline data and conducting propensity score matching. The findings confirm our first hypothesis: on average we found a positive health advantage for those who transitioned from receiving disability benefits to employment compared to those who remained on disability benefits. This group differed at baseline from those remaining on benefits (they were healthier, younger, better educated and were closer to the labour market), but controlling for these differences did not alter the result. The impact on mental health was approximately twice that on physical health. This is congruent with UK results from Flint et al., who found that transitions from permanent sickness to employment were associated with an improvement in mental wellbeing but whose sample included both those in receipt of disability benefits and those not (Flint et al., 2013). There was partial support for our second hypothesis – no impact on physical health was identified for those moving from receiving disability benefits to unemployment compared with those remaining on disability benefits, but there was an unanticipated positive impact on mental health for those transitioning to unemployment. Finally, the impact on health of those moving onto ESA from IB did not differ from those remaining on IB, supporting our third hypothesis. Recent region-level and qualitative studies have suggested a more negative impact (Barr et al., 2015a, Barr et al., 2015b, Garthwaite, 2014, Garthwaite, 2015, Patrick, 2011).

### 4.2 Strengths

The dynamic relationship between work, welfare and health affects a significant proportion of the UK working age population who receive disability benefits; our study makes a vital contribution to understanding a contested policy area faced by many welfare state countries. To achieve this, our research takes advantage of the Understanding Society survey – a high quality panel dataset with employment and welfare status information, validated measures of health (SF-12) (Jenkinson et al., 1997, Ware et al., 1996), a sufficient sample size to analyse rarely occurring transitions, and which is based on a representative population sample. Drawing on a theoretical schema, three transitions of interest along the continuum towards greater labour market participation were identified, and specific hypotheses were proposed associated with each one. Prior to analysis of the outcomes, careful deliberation was given to study design. We sought to imitate a randomised controlled trial in order to test the hypotheses within a counterfactual causal framework and minimise confounding. Sensitivity analyses to check the common trends assumption using pre-baseline data and application of propensity score matching supported the findings of the main difference-in-difference analyses. Unlike standard matching or stratification approaches, propensity score matching circumvents the problem of multidimensionality, allowing multiple confounding variables to be included within the constraints of the sample size of the survey dataset. The observed characteristics included in the propensity score were balanced between the treatment and control groups through the matching strategy, and avoided structural confounding – i.e. inferences were made based only on groups that were exchangeable. Alternative matching strategies were tested in order to ensure bias and residual variance was kept to a minimum, and alternative methods for calculating standard errors were found not to influence the results.

### 4.3 Limitations

Consideration should be given to the ways in which confounding, bias, and error may remain when interpreting our results. The analyses provide evidence for health behaving as a pre-outcome confounder, in keeping with other studies suggesting this shapes both health and employment trajectories (Beatty and Fothergill, 2011, Gebel and Voßemer, 2014, Kemp and Davidson, 2010, Ki et al., 2011, Schuring et al., 2011, Thomas et al., 2005, van der Noordt et al., 2014, van Rijn et al., 2014). Because health acts as an outcome and as a confounder, drawing definite conclusions regarding the causal effect of employment transitions on health is a difficult task, even when using longitudinal data and applying a robust causal inference framework to the study design as we have done. Those making the transition to employment in analysis 1 were younger, better educated, less deprived, closer to the labour market, and had better mental and physical health at baseline than those remaining on disability benefit. This was taken into account in the study design, but unobserved time-varying factors may still confound the effect. The survey data does not provide direct information on disability welfare assessments, but it is likely that most transitions to employment were made voluntarily, whereas (because greater financial insecurity is attached to unemployment) transitions to unemployment were made involuntarily by those found ‘fit for work’. The positive mental health impact of transitions to unemployment was unexpected and further research is needed to investigate this result. The findings from analysis 3 should be the most robust to health confounding, as the timing of the IB reassessment process was influenced by bureaucratic factors rather than health status (Kennedy, 2014).

Individuals within the population of interest could be excluded from the analyses because of survey non-response, attrition from participation at subsequent waves, or (because analyses were based on complete cases) individual missing data. If these led to a non-representative sample this may limit the external validity, and thus generalisability, of the findings. Differential attrition across waves within the treatment and comparison groups could lead to selection bias. If individuals with poorer health were more likely to leave the survey if they were in the control group rather than the treatment group, the effect size may be biased towards the null hypothesis, i.e. underestimate the treatment effect (or, if vice-versa, the results would bias towards an over-estimate). Both of these scenarios are plausible. Non-differential attrition across waves will reduce the sample size and may bias the results towards the null. Of the individuals in the survey with exposure data, the proportion of missing SF-12 health outcome data ranged from 23% to 33%. Most of the survey is carried out using a face-to-face computer aided interview, whereas the health outcome data (required at a minimum of two consecutive waves for inclusion in our sample) is administered by a self-completion questionnaire which survey participants may fail to return (Mcfall, 2013). However, analyses of other variables with no missing data (sex, age, education, marital status, long-standing illness status) did not suggest the exclusion of those with missing health outcome data changed the characteristics of the treatment and control groups (data not shown). It is likely that some misclassification involving variables such as the total disability count and educational level occurred, as well as exposure information which was based on self-reported employment status and welfare data. The extent of this problem is difficult to assess, but inconsistencies identified in the employment and welfare data affected small numbers.

The last wave of data collection used in this study took place in 2012/13, since then UK welfare policy for those receiving disability benefits has increased the use of financial penalties for non-compliance with benefit conditions (Work and Pensions Committee, 2015). This study has not looked at health trends over time, and our findings do not preclude the possibility that average health has worsened for those receiving disability benefits.

### 4.4 Implications for policy and research

Research exploring the questions generated by our study would be valuable in order to inform policy in this area. First, there is a clear need for explanations of the mechanisms that lie behind the relationship between work and health, specific to this group. The results from this study identify, on average, a beneficial health impact for transitions to employment. As with most studies using observational data and natural experiments, we did not have access to qualitative data that could illuminate our findings. The mechanisms underlying the positive relationship between work and health have been extensively debated in the research literature, and include reduced social stigma, reduced financial strain, increased material resources, and increased social capital (Backhans and Hemmingsson, 2012, Bambra, 2010, Janlert and Hammarstrom, 2009). However, these factors have largely been explored for transitions from unemployment and may differ amongst those receiving disability benefits transitioning to employment. Those with the worst health and who are most distant from the labour market are more likely to live in areas with the lowest job opportunities. Furthermore, the evidence regarding the effectiveness of return-to-work programmes in improving the employment chances of those with disabilities and chronic illnesses is unclear and it is difficult to attribute positive outcomes directly with the intervention because there have been few controlled studies (Ayala and Rodríguez, 2013, Bambra et al., 2005, Black, 2008, Huber et al., 2011) and rates of labour market return remain low – in contrast to the unemployed – particularly for those that previously received IB (CESI, 2015).

Second, existing literature suggests the quality of work individuals move into will modify health outcomes. Beyond return-to-work interventions it is important to consider access to training which will help this group move beyond the low pay sector, along with demand-side policies to help ensure employers create jobs and workplaces that will meet the needs of those with disabilities and long-term illness. Jobs that offer greater social status, social capital, financial security, and financial resources are likely to see a bigger improvement in health than poor quality jobs. Larger sample sizes are needed to explore this. Third, as further waves of Understanding Society data become available it should be possible to see if the health impacts persist or are transient, though the panel sample size will be reduced from attrition over longer periods. It would also be helpful to repeat the analyses with other measures of health, and directly compare the effect on different outcomes – we found a greater effect on mental health compared to physical health, but a larger sample size is needed to enable a formal statistical comparison. Beyond the data sources currently available, exploring these relationships using administrative data could be very fruitful as it would provide a large sample size, distinguish between the categories of ESA that have different levels of financial support and conditions attached, and if linked to health records and survey data could provide a rich set of health measures. Finally, it is important to highlight that for both treatment and control groups the health outcomes at baseline were well below the overall population mean mental and physical health score. Given the low rates of transition into employment, addressing the social and political determinants of population health in order to prevent the causes of health-related worklessness and reduce the numbers moving into disability benefit receipt must be a priority.

### 4.5 Conclusion

In Great Britain over 2.5 million people receive out-of-work disability benefits. Although the government has introduced welfare reforms and return-to-work programmes seeking to move this group back into the labour market it remains rare for individuals to return to active labour market participation. Our results indicate that on average such transitions may improve health, particularly mental health, although it is difficult to be certain on the direction of causality. Further research is needed to explore the findings from this study, for example, improving our understanding of the mechanisms that underlie the health impacts of differing employment conditions for this group, such as financial security and reduced stigma. Policy that integrates these factors into the welfare system for those remaining on disability benefits could help to ensure not only that ‘work is good for health’, but also that ‘welfare is good for health’.

## Figures and Tables

**Table 1:** Summary of mean SF-12 scores, DiD and DiD-PSM estimates.

	*Total n*	Mean SF-12 summary score by group						Difference-in-difference (DiD)					DiD with propensity score matching (DiD-PSM)					
Control (d0)			Treatment (d1)		
*n*	t0	t1	*n*	t0	t1	*Total n*	DiD	p-value	Lower CI	Upper CI	*Total n*	DiD-PSM	p-value	Lower CI	Upper CI	Off CS
**Analysis 1**																		
Mental	*1669*	*1545*	38.56	37.79	*124*	42.18	47.35	*1669*	5.94	<0.01	3.52	8.36	*1497*	5.63	<0.01	2.65	8.61	10
Physical	*1671*	*1547*	31.43	31.41	*124*	41.77	44.58	*1671*	2.83	0.01	0.85	4.81	*1499*	2.53	0.04	0.13	4.93	10
**Analysis 2**																		
Mental	*1698*	*1545*	38.56	37.79	*153*	38.10	40.47	*1698*	3.14	<0.01	1.17	5.11	*1527*	2.46	0.04	0.07	4.85	0
Physical	*1700*	*1547*	31.43	31.41	*153*	40.51	40.73	*1700*	0.24	0.80	−1.61	2.08	*1529*	1.38	0.20	−0.75	3.50	0
**Analysis 3**																		
Mental	*1285*	*1163*	39.55	38.48	*122*	36.87	36.23	*1285*	0.43	0.67	−1.51	2.36	*1145*	−0.10	0.92	−2.14	1.93	0
Physical	*1285*	*1163*	30.79	30.74	*122*	32.56	31.00	*1285*	−1.51	0.06	−3.10	0.08	*1145*	−1.01	0.26	−2.76	0.73	0

Notes: n = sample number, CI = 95% confidence interval, CS = Common Support, t0 = baseline, t1 = follow-up. DiD-PSM estimates were calculated for cases with complete covariate data only.
