# Supplementary material for: The impact on health of employment and welfare transitions for those receiving out-of-work disability benefits in the UK
Source: Soc Sci Med. 2016 Aug;162:1–10. doi: 10.1016/j.socscimed.2016.05.042 (PMC4962812; doi:10.1016/j.socscimed.2016.05.042)
Supplement: Supplementary file 1 [file mmc1.docx]

**The impact on health of employment and welfare transitions for those receiving out-of-work disability benefits in the UK: Web appendices**

Contents

[Appendix 1: Deriving transitions from Understanding Society data 3](#_Toc451855814)

[Appendix 2: Further detail on construction of exposure and outcome variables and covariates used in the propensity score 4](#_Toc451855815)

[Appendix 3: Numbers in each transition period & missing data 10](#_Toc451855816)

[Appendix 4: Mean SF-12 mental and physical score by baseline & follow-up status, treatment group status and analysis 12](#_Toc451855817)

[Appendix 5: Baseline health data for those with complete and missing propensity score covariate data 12](#_Toc451855818)

[Appendix 6: Summary of matching strategies 14](#_Toc451855819)

[Appendix 7: Balance of covariates at t0 between treatment and control groups before and after matching 15](#_Toc451855820)

[Appendix 8: Analysis One Propensity Score Matching (figures) 17](#_Toc451855821)

[Appendix 9: Analysis Two Propensity Score Matching (figures) 20](#_Toc451855822)

[Appendix 10: Analysis Three Propensity Score Matching (figures) 23](#_Toc451855823)

[Appendix 11: Using psmatch2 command with bias corrected bootstrapping for standard errors and teffects command 26](#_Toc451855824)

[Appendix 12: Analysis of pre-baseline trends 27](#_Toc451855825)

[Appendix 13: Treatment and control group baseline covariates 28](#_Toc451855826)

[Appendix 14: Summary of matching strategy, analysis 1 (pre-baseline sample only) 31](#_Toc451855827)

[Appendix 15: Balance of covariates at t-1 between treatment and control groups before and after matching (pre-baseline sample only) 31](#_Toc451855828)

[Appendix 16: DiD-PSM for analysis 1 (pre-baseline sample only) 32](#_Toc451855829)

[Web appendix References 33](#_Toc451855830)

### Appendix 1: Deriving transitions from Understanding Society data


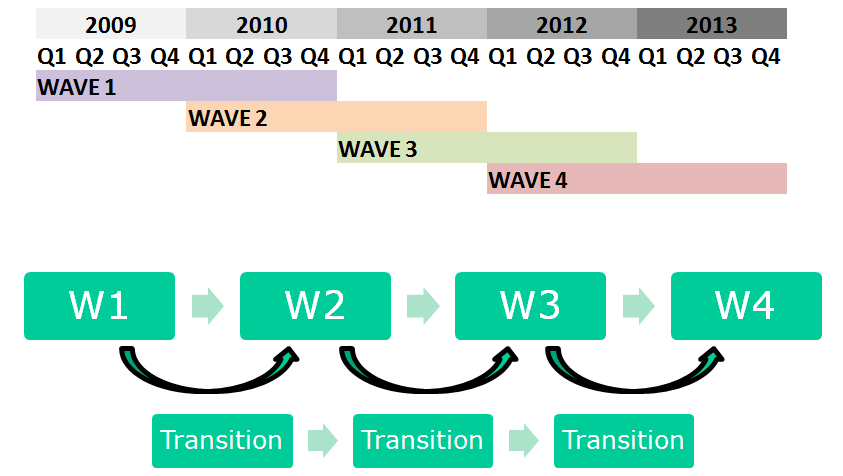


Note: Data for the main analyses presented utilised all three transition periods (w1 to w2, w2 to w3 and w3 to w4) to supply data for t0 to t1 intervention period data. Data for the sensitivity analyses using pre-baseline data required pre-baseline wave information, so only transitions from w2 to w3 and w3 to w4 could be used to supply t0 to t1 data; w1 to w2 and w2 to w3 could be used for pre-baseline transition data (t-1 to t0) if the individual also had follow up (t1) data.

### Appendix 2: Further detail on construction of exposure and outcome variables and covariates used in the propensity score

###### Construction of exposure variables

Self-reported employment status data, based on responses to the question ‘which of these best describes your current employment situation?’ was combined with information derived from self-reported benefit receipt to construct economic status groups. Incapacity benefit (IB), severe disablement allowance (SDA) and employment and support allowance (ESA) are all working age income-replacement disability benefits. Data from all three were combined for analyses one and two, but for analysis three ESA receipt was kept distinct. As with IB, recipients of SDA undergoing reassessment were transferred to ESA if they met the eligibility criteria. As only a very small number of people in the sample received SDA (it was abolished for new applicants in 2001) groups where this was combined with IB were simply labelled ‘IB’.

The employed category consists of those self-reporting their current employment situation as being in paid employment (full or part-time) or self-employed and who did not indicate receipt of disability benefits or unemployment benefits. The unemployed category consists of those self-reporting as unemployed plus those who indicated they received unemployment benefits, and who didn’t receive disability benefits. The disability benefit group consisted of all those stating they received disability benefits, regardless of their self-reported employment situation. In effect, when responses indicated their economic situation was unemployed or employed but receiving disability benefits (mutually exclusive categories in the schema used in the analysis), receiving disability benefit took preference. This decision was made because it was assessed that a risk of a ‘false positive’ response to the specific questions regarding benefit receipt status for IB or ESA was less likely than a ‘false positive’ to the more generic employment situation question which is more open to individual interpretation. Furthermore, up to 16 hours of paid work is permitted whilst receiving IB/ESA and this group might well describe themselves as employed.

When responses indicated individuals were receiving both ESA and IB, receiving ESA took preference. Because ESA has been introduced more recently it was thought there would greater clarity from respondents as to whether or not they were receiving this than IB. It is less likely those individuals who remained on IB and hadn’t been reassessed for ESA would incorrectly state they were receiving both, whereas it is feasible individuals who had transitioned onto ESA from IB after reassessment might potentially state incorrectly they were receiving both forms of disability benefit.

Additional sensitivity tests excluding the small number of individuals who appeared to work more than 16 hours per week at baseline whilst receiving disability benefits did not change findings when the Difference-in-Difference estimates were recalculated for each analysis (data submitted to reviewers, available on request).

###### Construction of outcome variables

The ‘Short Form-12’ (SF-12) was completed as a component of a self-completion questionnaire within the main survey of Understanding Society. This asks twelve questions relating to both physical and mental functioning (components summarised in box 1). Separate physical and mental summary score variables supplied within the survey are derived from responses by converting valid responses into a single score ranging from 0 (low functioning) to 100 (high functioning), with a mean population score of 50. Answers from a proxy respondent were excluded. The SF-12 has been tested for reliability and validity, and it has demonstrated that it accurately replicates results from the longer 36 item version of the health survey in longitudinal studies, including the physical and mental health summary scores on the 0-100 scale (Jenkinson & Layte, 1997; Jenkinson et al., 1997; Ware, Kosinski, & Keller, 1996).

Box 1: Components of the SF-12

- Self-rated health
- Health limits typical activities
- Health limits climbing stairs
- Physical health- accomplished less than you would like
- Physical health - limits kind of work or daily activities
- Emotional problems- accomplished less than would like
- Emotional problems – work/activities less carefully
- Pain – interferes with normal work (incl. housework)
- Felt calm & peaceful
- Had lot of energy
- Felt downhearted or depressed
- Physical health or emotional problems interfered with social activities

The lowest possible score on the physical summary scale indicates limitations in self-care, social and role activities, severe bodily pain, frequent tiredness and health rated “poor”. The highest possible score indicates no physical limitations, disabilities or decrements in well-being, high energy level, health rated “excellent”. The lowest possible score on the mental summary scale indicates frequent psychological distress, social and role disability due to emotional problems, health rated “poor”. The highest possible score indicates frequent positive affect, absence of psychological distress and limitations in usual social/role activities due to emotional problems, health rated “excellent” (Ware Jr, 2000).

###### The construction of covariates included in the propensity score

*Housing Tenure*

The household response datafiles in Understanding society include a derived housing tenure variable at each wave which assigns households into one of eight categories including those of ‘owned outright’ and ‘owned with mortgage’. These two categories where combined to create a binary housing tenure variable based on whether or not they fell into the house owner (outright or mortgage) category.

*Number of own children in household*

This was based on a derived variable in the individual response datafile which summarised the number of their own children in the household under age 16, including natural children, adopted children and step-children.

*Gross monthly income*

This was based on a derived variable in the individual response datafile. Individuals are asked to report wages, self-employment earnings, second job earnings, interest and dividends, pension income, benefits and other income sources such as rent from another property. These are summed to obtain the total personal income. Where data are missing for responding household the income variables are imputed using chained equations (ICE) in order to compute total personal income (Mcfall, 2013).

*Social Class*

This was based on a derived variable in the individual response datafile. First, a variable providing a five-category version of the National Statistics Socio-economic Classification (NS-SEC) based on responses to questions about their last job asked at wave 1 was included. This question was only asked of those that had not been in paid employment in the last week but were in paid employment in the past, and was not repeated at later waves unless they were a new entrant. The variable is constructed from data that indicates the type of occupation and their managerial duties, and assigns individuals to either 1 ‘Management & professional’, 2 ‘Intermediate’, 3 ‘Small employers & own account’, 4 ‘Lower supervisory & technical’, 5 ‘Semi-routine, routine & never worked/LT unemployed’. Those with missing data for this variable but who had indicated that they never had a job were added into category 5. Some of those with missing data had current job social class data and so this was used if data was missing.

*Years since last job (at first wave of survey participation)*

A variable ‘Years since last job’ was based on a derived variable in the individual response datafile, providing an indicator of how close the individual was to the labour market. First, a variable based on responses to a question about the year they left their last paid job was included. This was only asked of those who did no paid work in the last week and did not have a job but had indicated that they had had a job in the past, and was only asked at wave 1 unless they were a new entrant at later waves. Those with missing data but who indicated that they either worked in the last week or had a paid job (even if no work in the last week) were added to a new category ‘in paid work at first wave’, and another category was created for those who had never had a job. The year was re-calculated as ‘years since last job’ based on the year they responded to the survey. The final variable of years since last job had five categories: ‘In paid work at first wave’, ‘>0-10 years’, ‘11-20 years’, ‘>20 years’ and ‘never had a job’. It is important to note that the construction of the variable means that some falling into the category of ‘in paid work at first wave’ will at a later wave indicate they are receiving disability benefit (and potentially be included in one of the analyses control or treatment groups on the basis of a wave 2 to wave 3 transition, or a wave 3 to wave 4 transition), whereas others may fall into a treatment or control group based on a wave 1 to wave 2 transition. Therefore some individuals could indicate receiving disability benefits whilst at the same time being in some form of paid work. This group were presumed to fall under the permitted hours regulations (see main text section 1.1).

*Region*

This was based on a derived variable in the individual response datafile with 12 categories indicating government office region of residence. A simplified four-category version was created with the North-east, North-west, Yorkshire & the Humber, East midlands and West Midlands combined into the category ‘North England & Midlands’; East of England, London, South-east, South-west and Wales merged into ‘South England & Wales’; Scotland; Northern Ireland.

*Longstanding illness or disability*

Individuals were asked ‘Do you have any long-standing physical or mental impairment, illness or disability? By ‘long-standing I mean anything that has troubled you over a period of at least 12 months or that is likely to trouble you over a period of at least 12 months’. Responses were coded either ‘yes’ or ‘no’.

*Total disability count*

A count of self-reported disabilities was constructed from a series of variables that asked the participant if they had ‘substantial difficulties’ with specified areas; only participants who had indicated in a prior question that they had a long-standing illness or disability were asked these questions in the main survey. Areas covered were mobility, lifting/carrying, manual dexterity, continence, hearing, sight, communication problems, memory / learning, recognition of physical danger, physical coordination, personal care, and a final ‘other health problem or disability’. It was also possible to indicate ‘none of these’. A total count variable was created by summing the number of positive responses to any of these items. Those who were not asked the question because they had already indicated that they did not have a long-standing illness or disability were counted as zero.

*Wellbeing (GHQ)*

The ‘General Health Questionnaire’ (GHQ) was completed as a component of a self-completion questionnaire within the main survey. The GHQ is a twelve-item validated screening tool for identifying psychological distress. The derived variable used gives a score between 0 (least distressed) and 36 (most distressed) based on a summing of re-scaled item responses along a Likert-scale.

### Appendix 3: Numbers in each transition period & missing data

|  |  | Analysis 1 | |  | Analysis 2 | |  | Analysis 3 | |
| --- | --- | --- | --- | --- | --- | --- | --- | --- | --- |
|  |  | Treated | Control |  | Treated | Control |  | Treated | Control |
| **Total with transition data** | |  |  |  |  |  |  |  |  |
| Male | W1-W2 | 24 | 452 |  | 37 | 452 |  | 9 | 409 |
|  | W2-W3 | 21 | 321 |  | 35 | 321 |  | 16 | 267 |
|  | W3-W4 | 22 | 252 |  | 46 | 252 |  | 39 | 166 |
|  |  |  |  |  |  |  |  |  |  |
| Female | W1-W2 | 37 | 451 |  | 34 | 451 |  | 10 | 400 |
|  | W2-W3 | 33 | 370 |  | 40 | 370 |  | 46 | 265 |
|  | W3-W4 | 25 | 317 |  | 38 | 317 |  | 45 | 162 |
|  |  |  |  |  |  |  |  |  |  |
|  | Total (male & female) | 162 | 2163 |  | 230 | 2163 |  | 165 | 1669 |
|  |  |  |  |  |  |  |  |  |  |
| **Total with t0 & t1 health data** | |  |  |  |  |  |  |  |  |
| Mental health | N with t0 health data | 151 | 1882 |  | 193 | 1882 |  | 137 | 1449 |
|  | *% missing t0* | *7* | *13* |  | *16* | *13* |  | *17* | *13* |
|  | N with t1 health data | 130 | 1749 |  | 173 | 1749 |  | 142 | 1315 |
|  | *% missing t1* | *20* | *19* |  | *25* | *19* |  | *14* | *21* |
|  | *Total with t0 & t1 data* | *124* | *1545* |  | *153* | *1545* |  | *122* | *1163* |
|  | *Total % missing* | *23* | *29* |  | *33* | *29* |  | *26* | *30* |
|  |  |  |  |  |  |  |  |  |  |
| Physical health | N with t0 health data | 151 | 1884 |  | 193 | 1884 |  | 137 | 1449 |
|  | *% missing t1* | *7* | *13* |  | *16* | *13* |  | *17* | *13* |
|  | N with t1 health data | 130 | 1750 |  | 173 | 1750 |  | 142 | 1315 |
|  | *% missing t2* | *20* | *19* |  | *25* | *19* |  | *14* | *21* |
|  | *Total with t0 & t1 data* | *124* | *1547* |  | *153* | *1547* |  | *122* | *1163* |
|  | *Total % missing* | *23* | *28* |  | *33* | *28* |  | *26* | *30* |
|  |  |  |  |  |  |  |  |  |  |
| **Percentage missing for potential propensity score co-variables** | | | |  |  |  |  |  |  |
|  | Age | 0 | 0 |  | 0 | 0 |  | 0 | 0 |
|  | Sex | 0 | 0 |  | 0 | 0 |  | 0 | 0 |
|  | Education | 0 | 0 |  | 0 | 0 |  | 0 | 0 |
|  | Marital status | 0 | 0 |  | 0 | 0 |  | 0 | 0 |
|  | Region | 0 | 0 |  | 0 | 0 |  | 0 | 0 |
|  | GHQ | 4 | 6 |  | 6 | 6 |  | 1 | 7 |
|  | Total disability count | 0 | 0 |  | 0 | 0 |  | 0 | 0 |
|  | Longstanding illness or disability | 0 | 0 |  | 0 | 0 |  | 0 | 0 |
|  | Time since last job | 0 | 4 |  | 3 | 4 |  | 2 | 5 |
|  | Social class | 0 | 3 |  | 3 | 3 |  | 0 | 4 |
|  | Housing tenure | 0 | 0 |  | 0 | 0 |  | 0 | 0 |
|  | Born in the UK | 0 | 0 |  | 0 | 0 |  | 0 | 0 |
|  | Number of own children in household | 0 | 0 |  | 0 | 0 |  | 0 | 0 |
|  | Gross monthly income (£) | 0 | 0 |  | 0 | 0 |  | 0 | 0 |

### Appendix 4: Mean SF-12 mental and physical score by baseline & follow-up status, treatment group status and analysis

|  |  | Baseline health (t0) | | | | | | |  |  | Follow up health (t1) | | | | | | |
| --- | --- | --- | --- | --- | --- | --- | --- | --- | --- | --- | --- | --- | --- | --- | --- | --- | --- |
|  | Follow up data (t1) | Treated (d1) | | |  | Control (d0) | | |  | Baseline data (t0) | Treated (d1) | | |  | Control (d0) | | |
|  |  | *n* | Mental | Physical |  | *n* | Mental | Physical |  |  | *n* | Mental | Physical |  | *n* | Mental | Physical |
| **Analysis 1** | |  |  |  |  |  |  |  |  |  |  |  |  |  |  |  |  |
|  | Yes | *124* | 42.2 | 41.8 |  | *1545* | 38.6 | 31.4 |  | Yes | *124* | 47.4 | 44.6 |  | *1545* | 37.8 | 31.4 |
|  | No | *27* | 43.5 | 44.8 |  | *337* | 37.9 | 31.8 |  | No | *6* | 51.0 | 46.1 |  | *204* | 36.0 | 33.2 |
| **Analysis 2** | |  |  |  |  |  |  |  |  |  |  |  |  |  |  |  |  |
|  | Yes | *153* | 38.1 | 40.5 |  | *1545* | 38.6 | 31.4 |  | Yes | *153* | 40.5 | 40.7 |  | *1545* | 37.8 | 31.4 |
|  | No | *30* | 39.9 | 37.7 |  | *337* | 37.9 | 31.8 |  | No | *20* | 39.4 | 39.0 |  | *204* | 36.0 | 33.2 |
| **Analysis 3** | |  |  |  |  |  |  |  |  |  |  |  |  |  |  |  |  |
|  | Yes | *122* | 36.9 | 32.6 |  | *1163* | 39.6 | 30.8 |  | Yes | *122* | 36.2 | 31.0 |  | *1163* | 38.5 | 30.7 |
|  | No | *15* | 34.5 | 33.2 |  | *286* | 38.6 | 31.7 |  | No | *20* | 31.9 | 31.3 |  | *152* | 37.6 | 33.2 |

### Appendix 5: Baseline health data for those with complete and missing propensity score covariate data

|  |  |  |  |  |  | Mean SF-12 summary health scores at baseline (t0) | | | | | | |
| --- | --- | --- | --- | --- | --- | --- | --- | --- | --- | --- | --- | --- |
|  | PSM covariate data |  | Sample (d1 & d0) | |  | Treated (d1) | | |  | Control (d0) | | |
|  |  |  | n | % |  | *n* | Mental | Physical |  | *n* | Mental | Physical |
| **Analysis 1** | |  |  |  |  |  |  |  |  |  |  |  |
|  | Complete |  | 1497 | 89.7 |  | *109* | 42.5 | 40.5 |  | *1388* | 38.5 | 31.4 |
|  | Missing |  | 172 | 10.3 |  | *15* | 40.0 | 51.0 |  | *157* | 38.8 | 31.3 |
|  | *Total n* |  | *1669* |  |  | *124* |  |  |  | *1545* |  |  |
| **Analysis 2** | |  |  |  |  |  |  |  |  |  |  |  |
|  | Complete |  | 1527 | 89.9 |  | *139* | 38.2 | 40.2 |  | *1388* | 38.5 | 31.4 |
|  | Missing |  | 171 | 10.1 |  | *14* | 37.3 | 44.0 |  | *157* | 38.8 | 31.3 |
|  | *Total n* |  | *1698* |  |  | *153* |  |  |  | *1545* |  |  |
| **Analysis 3** | |  |  |  |  |  |  |  |  |  |  |  |
|  | Complete |  | 1145 | 89.1 |  | *119* | 37.0 | 32.6 |  | *1026* | 39.5 | 30.7 |
|  | Missing |  | 140 | 10.9 |  | *3* | 33.5 | 30.1 |  | *137* | 40.1 | 31.3 |
|  | *Total n* |  | *1285* |  |  | *122* |  |  |  | *1163* |  |  |

### Appendix 6: Summary of matching strategies

|  | *Sample type* | *Off common-support* | *Mean bias (%)* | *Median bias (%)* | *Rubins' B (%)* | *Rubins' R* |
| --- | --- | --- | --- | --- | --- | --- |
| Analysis One | Original sample | n/a | 29.7 | 22.0 | 142.8 | 1.56 |
|  | Nearest neighbour 1:1 | n/a | 11.4 | 11.7 | 56.2 | 1.23 |
|  | Caliper (0.03) 1:1 | 10 | 9.6 | 8.7 | 55.3 | 1.16 |
|  | Caliper (0.03) 1:3 | 10 | 4.9 | 4.3 | 29.2 | 0.91 |
|  | Kernel (epan, bw=0.06) | 5 | 5.9 | 4.1 | 29.0 | 1.09 |
|  | **Kernel (epan, bw=0.03)** | **10** | **3.7** | **3.1** | **19.9** | **0.86** |
|  | Kernel (epan, bw=0.01) | 11 | 4.7 | 4.3 | 26.1 | 0.87 |
|  | Kernel (normal, bw=0.03) | 0 | 7.1 | 4.1 | 36.6 | 0.97 |
|  | Kernel (biweight) | 10 | 3.7 | 2.7 | 21.1 | 0.83 |
|  | LLR (epan, bw=0.03) | 0 | 11.4 | 11.7 | 56.2 | 1.23 |
|  | LLR (biweight, bw=0.03) | 10 | 3.7 | 2.4 | 22.1 | 0.82 |
|  |  |  |  |  |  |  |
|  |  |  |  |  |  |  |
| Analysis Two | Original sample | n/a | 22.0 | 17.0 | 101.9 | 1.05 |
|  | Nearest neighbour 1:1 | n/a | 6.3 | 4.1 | 38.5 | 0.99 |
|  | Caliper (0.02) 1:1 | 0 | 6.3 | 4.1 | 38.5 | 0.99 |
|  | Caliper (0.02) 1:3 | 0 | 2.9 | 2.0 | 22.3 | 1.39 |
|  | Kernel (epan, bw=0.06) | 0 | 2.0 | 1.5 | 9.8 | 1.19 |
|  | **Kernel (epan, bw=0.03)** | **0** | **1.8** | **1.5** | **9.4** | **1.09** |
|  | Kernel (epan, bw=0.01) | 1 | 2.2 | 1.9 | 11.8 | 1.05 |
|  |  |  |  |  |  |  |
| Analysis Three | Original sample | n/a | 11.6 | 10.0 | 50.2 | 1.11 |
|  | Nearest neighbour 1:1 | n/a | 7.3 | 7.4 | 40.3 | 0.96 |
|  | Caliper (0.01) 1:1 | 0 | 7.3 | 7.4 | 40.3 | 0.96 |
|  | Caliper (0.01) 1:3 | 0 | 4.7 | 3.1 | 27.3 | 0.91 |
|  | Kernel (epan, bw=0.06) | 0 | 2.8 | 2.2 | 13.0 | 1.19 |
|  | **Kernel (epan, bw=0.03)** | **0** | **1.4** | **1.2** | **6.9** | **0.96** |
|  | Kernel (epan, bw=0.01) | 0 | 1.9 | 1.8 | 9.5 | 0.85 |

Notes: CS = Common Support, bw = bandwidth, epan=epanechnikov (kernel type); selected strategy has values highlighted in bold. Rubin’s B is the absolute standardised difference (%) of the means of the linear index of the propensity score in the treatment and the comparison group after matching. Rubin’s R is the ratio of the treated to the control’s variances of the propensity score index after matching. The user-written Stata programme pstest suggests aiming to ensure Rubin’s B should be <25 and Rubin’s R between 0.5 and 2 for assessing the quality of the matching strategy, based on (Rosenbaum & Rubin, 1985) and (Rubin, 2001).

### Appendix 7: Balance of covariates at t0 between treatment and control groups before and after matching

|  |  | Analysis 1 | | |  | Analysis 2 | | |  | Analysis 3 | | |
| --- | --- | --- | --- | --- | --- | --- | --- | --- | --- | --- | --- | --- |
|  |  | Treated | Control | %bias |  | Treated | Control | %bias |  | Treated | Control | %bias |
|  |  | (Kernel, bw 0.03) | | |  | (Kernel, bw 0.03) | | |  | (Kernel, bw 0.03) | | |
|  |  |  |  |  |  |  |  |  |  |  |  |  |
| Sex (Ref: male) | Before matching | 0.62 | 0.54 | 17.0 |  | 0.52 | 0.54 | -4.2 |  | 0.60 | 0.50 | 18.9 |
|  | After matching | 0.60 | 0.59 | 0.5 |  | 0.50 | 0.51 | -1.4 |  | 0.60 | 0.60 | -0.6 |
|  |  |  |  |  |  |  |  |  |  |  |  |  |
| Age in years | Before matching | 42.43 | 48.07 | -52.1 |  | 41.35 | 48.07 | -62.3 |  | 46.89 | 49.39 | -25.6 |
|  | After matching | 43.26 | 41.96 | 12.0 |  | 41.75 | 41.45 | 2.8 |  | 46.82 | 47.13 | -3.2 |
|  |  |  |  |  |  |  |  |  |  |  |  |  |
| Marital status (Ref: not married/civil p'ship) | Before matching | 0.45 | 0.42 | 6.0 |  | 0.28 | 0.42 | -29.8 |  | 0.39 | 0.45 | -12.5 |
|  | After matching | 0.46 | 0.44 | 3.9 |  | 0.27 | 0.27 | 0.6 |  | 0.39 | 0.38 | 0.5 |
|  |  |  |  |  |  |  |  |  |  |  |  |  |
| Region: N.England & Midlands | Before matching | 0.35 | 0.42 | -14.5 |  | 0.44 | 0.42 | 5.5 |  | 0.43 | 0.41 | 5.4 |
|  | After matching | 0.36 | 0.37 | -3.3 |  | 0.45 | 0.46 | -3.6 |  | 0.43 | 0.42 | 2.1 |
|  |  |  |  |  |  |  |  |  |  |  |  |  |
| Region: S.England & Wales | Before matching | 0.56 | 0.43 | 25.6 |  | 0.42 | 0.43 | -2.2 |  | 0.41 | 0.43 | -3.1 |
|  | After matching | 0.55 | 0.55 | 0.6 |  | 0.42 | 0.41 | 3.9 |  | 0.41 | 0.41 | -0.1 |
|  |  |  |  |  |  |  |  |  |  |  |  |  |
| Region: Scotland | Before matching | 0.06 | 0.09 | -10.0 |  | 0.10 | 0.09 | 4.5 |  | 0.12 | 0.09 | 10.5 |
|  | After matching | 0.06 | 0.05 | 4.8 |  | 0.11 | 0.11 | -0.8 |  | 0.13 | 0.13 | -2.3 |
|  |  |  |  |  |  |  |  |  |  |  |  |  |
| Region: N.Ireland | Before matching | 0.03 | 0.06 | -14.1 |  | 0.03 | 0.06 | -13.9 |  | 0.03 | 0.08 | -19.3 |
|  | After matching | 0.03 | 0.03 | 0.1 |  | 0.02 | 0.02 | 0.5 |  | 0.03 | 0.04 | -1.2 |
|  |  |  |  |  |  |  |  |  |  |  |  |  |
| Degree education (Ref: A-Level or lower) | Before matching | 0.30 | 0.20 | 24.0 |  | 0.10 | 0.20 | -27.8 |  | 0.20 | 0.20 | 2.4 |
|  | After matching | 0.28 | 0.25 | 5.0 |  | 0.10 | 0.10 | -0.4 |  | 0.21 | 0.21 | -1.1 |
|  |  |  |  |  |  |  |  |  |  |  |  |  |
| Long-standing illness or disability (Ref: Yes) | Before matching | 0.20 | 0.05 | 46.9 |  | 0.18 | 0.05 | 42.3 |  | 0.07 | 0.04 | 10.0 |
|  | After matching | 0.17 | 0.14 | 6.4 |  | 0.19 | 0.19 | 2.5 |  | 0.07 | 0.07 | 0.8 |
|  |  |  |  |  |  |  |  |  |  |  |  |  |
| General Health Questionnaire (0-36) | Before matching | 16.06 | 17.72 | -21.1 |  | 17.30 | 17.72 | -5.5 |  | 18.54 | 17.12 | 18.5 |
|  | After matching | 16.41 | 16.30 | 1.4 |  | 17.14 | 17.34 | -2.7 |  | 18.44 | 18.46 | -0.3 |
|  |  |  |  |  |  |  |  |  |  |  |  |  |
| Total disability count (0-12) | Before matching | 1.77 | 3.45 | -76.2 |  | 2.03 | 3.45 | -65.5 |  | 3.31 | 3.53 | -9.3 |
|  | After matching | 1.91 | 1.94 | -1.6 |  | 2.06 | 2.10 | -1.5 |  | 3.29 | 3.33 | -1.7 |
|  |  |  |  |  |  |  |  |  |  |  |  |  |
| Gross monthly income (£) | Before matching | 1321.90 | 1122.30 | 22.0 |  | 1090.10 | 1122.30 | -4.6 |  | 1171.60 | 1113.50 | 8.3 |
|  | After matching | 1265.30 | 1234.50 | 3.4 |  | 1086.00 | 1102.00 | -2.3 |  | 1175.10 | 1184.20 | -1.3 |
|  |  |  |  |  |  |  |  |  |  |  |  |  |
| Number of own children in household | Before matching | 0.48 | 0.37 | 12.8 |  | 0.57 | 0.37 | 21.2 |  | 0.37 | 0.32 | 6.1 |
|  | After matching | 0.50 | 0.57 | -6.9 |  | 0.55 | 0.55 | -0.8 |  | 0.37 | 0.36 | 1.3 |
|  |  |  |  |  |  |  |  |  |  |  |  |  |
| UK born (ref=no) | Before matching | 0.90 | 0.91 | -1.4 |  | 0.88 | 0.91 | -10.1 |  | 0.93 | 0.91 | 5.7 |
|  | After matching | 0.91 | 0.92 | -3.1 |  | 0.88 | 0.90 | -4.1 |  | 0.92 | 0.92 | 0.7 |
|  |  |  |  |  |  |  |  |  |  |  |  |  |
| House owner or mortgage (ref=no) | Before matching | 0.53 | 0.41 | 24.4 |  | 0.18 | 0.41 | -53.2 |  | 0.35 | 0.45 | -20.9 |
|  | After matching | 0.51 | 0.49 | 4.6 |  | 0.18 | 0.18 | -0.6 |  | 0.35 | 0.36 | -2.3 |
|  |  |  |  |  |  |  |  |  |  |  |  |  |
| In paid work at first wave | Before matching | 0.49 | 0.08 | 102.7 |  | 0.08 | 0.08 | 1.0 |  | 0.03 | 0.03 | -5.0 |
|  | After matching | 0.44 | 0.43 | 2.3 |  | 0.09 | 0.10 | -3.3 |  | 0.03 | 0.03 | -2.5 |
|  |  |  |  |  |  |  |  |  |  |  |  |  |
| Years since last job: <1 year to 10 years | Before matching | 0.39 | 0.49 | -21.2 |  | 0.62 | 0.49 | 26.3 |  | 0.55 | 0.49 | 13.0 |
|  | After matching | 0.42 | 0.42 | 0.7 |  | 0.63 | 0.62 | 1.7 |  | 0.55 | 0.56 | -0.7 |
|  |  |  |  |  |  |  |  |  |  |  |  |  |
| Years since last job: 11 to 20 years | Before matching | 0.07 | 0.28 | -56.9 |  | 0.16 | 0.28 | -29.0 |  | 0.25 | 0.33 | -17.5 |
|  | After matching | 0.08 | 0.09 | -2.4 |  | 0.17 | 0.16 | 1.0 |  | 0.24 | 0.25 | -1.2 |
|  |  |  |  |  |  |  |  |  |  |  |  |  |
| Years since last job: >20 years | Before matching | 0.02 | 0.08 | -30.5 |  | 0.04 | 0.08 | -17.0 |  | 0.07 | 0.09 | -9.7 |
|  | After matching | 0.02 | 0.02 | -1.2 |  | 0.04 | 0.04 | 0.1 |  | 0.07 | 0.07 | 0.4 |
|  |  |  |  |  |  |  |  |  |  |  |  |  |
| Years since last job: never had a job | Before matching | 0.03 | 0.07 | -16.0 |  | 0.09 | 0.07 | 10.2 |  | 0.11 | 0.06 | 17.6 |
|  | After matching | 0.04 | 0.04 | -0.7 |  | 0.08 | 0.08 | -1.4 |  | 0.11 | 0.10 | 4.3 |
|  |  |  |  |  |  |  |  |  |  |  |  |  |
| NS-SEC (1-5) | Before matching | 3.23 | 3.71 | -29.2 |  | 4.11 | 3.71 | 26.8 |  | 3.80 | 3.71 | 5.3 |
|  | After matching | 3.31 | 3.52 | -12.5 |  | 4.08 | 4.13 | -3.1 |  | 3.83 | 3.82 | 0.7 |

### Appendix 8: Analysis One Propensity Score Matching (figures)

###### Density graph before matching

###### Density graph after matching

###### Common Support

###### Bias before & after matching across covariates

###### Bias against variance, before and after matching

### Appendix 9: Analysis Two Propensity Score Matching (figures)

###### Density graph before matching

###### Density graph after matching

###### Common Support

###### Bias before & after matching across covariates

###### Bias against variance, before and after matching

### Appendix 10: Analysis Three Propensity Score Matching (figures)

###### Density graph before matching

###### Density graph after matching

###### Common Support

###### Bias before & after matching across covariates

###### Bias against variance, before and after matching

### Appendix 11: Using psmatch2 command with bias corrected bootstrapping for standard errors and teffects command

|  | DiD with Propensity Score Matching (DiD-PSM) | | | | | |  | DID-PSM with bias-corrected bootstrapping (psmatch2)* | | | |  | DID-PSM with 3 nearest neighbour matching & robust Abadie-Imbens standard errors (teffects)^ | | | | |
| --- | --- | --- | --- | --- | --- | --- | --- | --- | --- | --- | --- | --- | --- | --- | --- | --- | --- |
|  |  |  |  |  |  |  |  |  |  |  |  |  |  |  |  |  |  |
|  | *n* | DiD-PSM | p-value | Lower CI | Upper CI | off CS |  | *n* | DID-PSM | Lower CI | Upper CI |  | *n* | DID-PSM | p-value | Lower CI | Upper CI |
| **Analysis 1** |  |  |  |  |  |  |  |  |  |  |  |  |  |  |  |  |  |
|  |  |  |  |  |  |  |  |  |  |  |  |  |  |  |  |  |  |
| Mental health | *1497* | 5.63 | <0.001 | 2.65 | 8.61 | 10 |  | *1497* | 5.63 | 2.58 | 8.51 |  | *1497* | 7.20 | <0.001 | 4.22 | 10.18 |
| Physical health | *1499* | 2.53 | 0.039 | 0.13 | 4.93 | 10 |  | *1499* | 2.53 | 0.52 | 4.90 |  | *1499* | 2.40 | 0.026 | 0.29 | 4.50 |
|  |  |  |  |  |  |  |  |  |  |  |  |  |  |  |  |  |  |
| **Analysis 2** |  |  |  |  |  |  |  |  |  |  |  |  |  |  |  |  |  |
|  |  |  |  |  |  |  |  |  |  |  |  |  |  |  |  |  |  |
| Mental health | *1527* | 2.46 | 0.044 | 0.07 | 4.85 | 0 |  | *1527* | 2.46 | -0.07 | 4.67 |  | *1527* | 2.56 | 0.052 | -0.02 | 5.14 |
| Physical health | *1529* | 1.38 | 0.204 | -0.75 | 3.50 | 0 |  | *1529* | 1.38 | -0.52 | 3.30 |  | *1529* | 0.85 | 0.426 | -1.24 | 2.94 |
|  |  |  |  |  |  |  |  |  |  |  |  |  |  |  |  |  |  |
| **Analysis 3** |  |  |  |  |  |  |  |  |  |  |  |  |  |  |  |  |  |
|  |  |  |  |  |  |  |  |  |  |  |  |  |  |  |  |  |  |
| Mental health | *1145* | -0.10 | 0.922 | -2.14 | 1.93 | 0 |  | *1145* | -0.10 | -1.95 | 2.11 |  | *1145* | 0.04 | 0.973 | -2.40 | 2.48 |
| Physical | *1145* | -1.01 | 0.256 | -2.76 | 0.73 | 0 |  | *1145* | -1.01 | -2.61 | 1.08 |  | *1145* | -1.43 | 0.155 | -3.39 | 0.54 |
|  |  |  |  |  |  |  |  |  |  |  |  |  |  |  |  |  |  |
| *200 replications, with adjustment for clusters on pidp | | | | | |  |  |  |  |  |  |  |  |  |  |  |  |
| ^restricted to same population that have common support as in main DID-PSM analysis | | | | | | | | | |  |  |  |  |  |  |  |  |

|  |  |  |  |  |  |  |  |  |  |  |  |  |
| --- | --- | --- | --- | --- | --- | --- | --- | --- | --- | --- | --- | --- |

### Appendix 12: Analysis of pre-baseline trends

|  |  |  | Mean SF-12 summary score by group | | | | | | | | |  | t-1 to t0 difference-in-difference (DID-prior trend) | | | | |  | t0 to t1 difference-in-difference (DID) | | | | |
| --- | --- | --- | --- | --- | --- | --- | --- | --- | --- | --- | --- | --- | --- | --- | --- | --- | --- | --- | --- | --- | --- | --- | --- |
|  |  |  | Control (d0) | | | |  | Treatment (d1) | | | |  |  |  |  |  |  |  |  |  |  |  |  |
|  | *Total n* |  | *n* | t-1 | t0 | t1 |  | *n* | t-1 | t0 | t1 |  | *Total n* | DID | p-value | Lower CI | Upper CI |  | *Total n* | DID | p-value | Lower CI | Upper CI |
| **Analysis 1** |  |  |  |  |  |  |  |  |  |  |  |  |  |  |  |  |  |  |  |  |  |  |  |
|  |  |  |  |  |  |  |  |  |  |  |  |  |  |  |  |  |  |  |  |  |  |  |  |
| Mental | *853* |  | *779* | 39.10 | 37.57 | 37.59 |  | *74* | 43.00 | 41.10 | 47.44 |  | *853* | -0.37 | 0.82 | -3.59 | 2.84 |  | *853* | **6.32** | <0.01 | 3.17 | 9.48 |
| Physical | *855* |  | *781* | 31.95 | 31.26 | 31.34 |  | *74* | 43.39 | 42.67 | 45.19 |  | *855* | -0.03 | 0.98 | -2.39 | 2.32 |  | *855* | 2.44 | 0.07 | -0.18 | 5.06 |
|  |  |  |  |  |  |  |  |  |  |  |  |  |  |  |  |  |  |  |  |  |  |  |  |
| **Analysis 2** |  |  |  |  |  |  |  |  |  |  |  |  |  |  |  |  |  |  |  |  |  |  |  |
|  |  |  |  |  |  |  |  |  |  |  |  |  |  |  |  |  |  |  |  |  |  |  |  |
| Mental | *871* |  | *779* | 39.10 | 37.57 | 37.59 |  | *92* | 41.10 | 37.46 | 39.43 |  | *871* | -2.11 | 0.12 | -4.79 | 0.58 |  | *871* | 1.95 | 0.12 | -0.50 | 4.40 |
| Physical | *873* |  | *781* | 31.95 | 31.26 | 31.34 |  | *92* | 41.16 | 39.76 | 40.51 |  | *873* | -0.72 | 0.56 | -3.14 | 1.70 |  | *873* | 0.67 | 0.60 | -1.83 | 3.18 |
|  |  |  |  |  |  |  |  |  |  |  |  |  |  |  |  |  |  |  |  |  |  |  |  |
| **Analysis 3** |  |  |  |  |  |  |  |  |  |  |  |  |  |  |  |  |  |  |  |  |  |  |  |
|  |  |  |  |  |  |  |  |  |  |  |  |  |  |  |  |  |  |  |  |  |  |  |  |
| Mental | *614* |  | *519* | 39.90 | 38.32 | 38.61 |  | *95* | 35.75 | 35.81 | 34.79 |  | *614* | 1.64 | 0.15 | -0.59 | 3.88 |  | *614* | -1.32 | 0.22 | -3.44 | 0.80 |
| Physical | *614* |  | *519* | 30.74 | 30.63 | 30.60 |  | *95* | 31.63 | 32.11 | 31.57 |  | *614* | 0.59 | 0.56 | -1.41 | 2.59 |  | *614* | -0.51 | 0.60 | -2.39 | 1.38 |

### Appendix 13: Treatment and control group baseline covariates

|  |  | **Remain on IB/ESA** | |  | **IB/ESA to employment** | |  | **IB/ESA to unemployment** | |  | **Remain on IB** | |  | **IB to ESA** | |
| --- | --- | --- | --- | --- | --- | --- | --- | --- | --- | --- | --- | --- | --- | --- | --- |
|  |  | **n** | **mean** |  | **n** | **mean** |  | **n** | **mean** |  | **n** | **mean** |  | **n** | **mean** |
| **Age** |  | 1545 | 48.1 |  | 124 | 42.4 |  | 153 | 41.3 |  | 1163 | 49.4 |  | 122 | 46.9 |
|  |  |  |  |  |  |  |  |  |  |  |  |  |  |  |  |
| **GHQ** |  | 1458 | 17.7 |  | 119 | 16.1 |  | 144 | 17.3 |  | 1085 | 17.1 |  | 121 | 18.5 |
|  |  |  |  |  |  |  |  |  |  |  |  |  |  |  |  |
| **Gross monthly income (£)** | | 1545 | 1122.3 |  | 124 | 1321.9 |  | 153 | 1090.1 |  | 1163 | 1113.5 |  | 122 | 1171.6 |
|  |  |  |  |  |  |  |  |  |  |  |  |  |  |  |  |
| **Net monthly income (£)** | | 1545 | 1111.7 |  | 124 | 1218.2 |  | 153 | 1071.7 |  | 1163 | 1104.2 |  | 122 | 1171.2 |
|  |  |  |  |  |  |  |  |  |  |  |  |  |  |  |  |
| **Total disability count*** | | 1545 | 3.5 |  | 124 | 1.8 |  | 153 | 2.0 |  | 1163 | 3.5 |  | 122 | 3.3 |
|  |  |  |  |  |  |  |  |  |  |  |  |  |  |  |  |
| **Number of own children in household** | | 1545 | 0.4 |  | 124 | 0.5 |  | 153 | 0.6 |  | 1163 | 0.3 |  | 122 | 0.4 |
|  |  |  |  |  |  |  |  |  |  |  |  |  |  |  |  |
| **Gender** |  | **n** | **%** |  | **n** | **%** |  | **n** | **%** |  | **n** | **%** |  | **n** | **%** |
|  | Male | 715 | 46.3 |  | 47 | 37.9 |  | 74 | 48.4 |  | 576 | 49.5 |  | 49 | 40.2 |
|  | Female | 830 | 53.7 |  | 77 | 62.1 |  | 79 | 51.6 |  | 587 | 50.5 |  | 73 | 59.8 |
|  | *Total n* | *1545* |  |  | *124* |  |  | *153* |  |  | *1163* |  |  | *122* |  |
| **Education** |  |  |  |  |  |  |  |  |  |  |  |  |  |  |  |
|  | Degree | 141 | 9.1 |  | 22 | 17.7 |  | 7 | 4.6 |  | 103 | 8.9 |  | 16 | 13.1 |
|  | Other higher degree | 161 | 10.4 |  | 15 | 12.1 |  | 8 | 5.2 |  | 124 | 10.7 |  | 9 | 7.4 |
|  | A-level etc | 233 | 15.1 |  | 27 | 21.8 |  | 23 | 15.0 |  | 160 | 13.8 |  | 19 | 15.6 |
|  | GCSE etc | 392 | 25.4 |  | 28 | 22.6 |  | 42 | 27.5 |  | 279 | 24.0 |  | 33 | 27.0 |
|  | Other qualification | 240 | 15.5 |  | 20 | 16.1 |  | 30 | 19.6 |  | 185 | 15.9 |  | 18 | 14.8 |
|  | No qualification | 378 | 24.5 |  | 12 | 9.7 |  | 43 | 28.1 |  | 312 | 26.8 |  | 27 | 22.1 |
|  | *Total n* | *1545* |  |  | *124* |  |  | *153* |  |  | *1163* |  |  | *122* |  |
| **Marital status** | |  |  |  |  |  |  |  |  |  |  |  |  |  |  |
|  | Not married / civil p'ship | 893 | 57.8 |  | 68 | 54.8 |  | 110 | 71.9 |  | 643 | 55.3 |  | 75 | 61.5 |
|  | Married / civil p'ship | 652 | 42.2 |  | 56 | 45.2 |  | 43 | 28.1 |  | 520 | 44.7 |  | 47 | 38.5 |
|  | *Total n* | *1545* |  |  | *124* |  |  | *153* |  |  | *1163* |  |  | *122* |  |
| **Long Standing Illness or Disability** | |  |  |  |  |  |  |  |  |  |  |  |  |  |  |
|  | Yes | 1468 | 95.0 |  | 99 | 79.8 |  | 125 | 81.7 |  | 1113 | 95.7 |  | 114 | 93.4 |
|  | No | 77 | 5.0 |  | 25 | 20.2 |  | 28 | 18.3 |  | 50 | 4.3 |  | 8 | 6.6 |
|  | *Total n* | *1545* |  |  | *124* |  |  | *153* |  |  | *1163* |  |  | *122* |  |
| **Region** |  |  |  |  |  |  |  |  |  |  |  |  |  |  |  |
|  | N.England & Midlands | 644 | 41.7 |  | 43 | 34.7 |  | 68 | 44.4 |  | 474 | 40.8 |  | 53 | 43.4 |
|  | S.England & Wales | 663 | 42.9 |  | 69 | 55.6 |  | 64 | 41.8 |  | 494 | 42.5 |  | 50 | 41.0 |
|  | Scotland | 141 | 9.1 |  | 8 | 6.5 |  | 16 | 10.5 |  | 105 | 9.0 |  | 15 | 12.3 |
|  | N.Ireland | 96 | 6.2 |  | 4 | 3.2 |  | 5 | 3.3 |  | 89 | 7.7 |  | 4 | 3.3 |
|  | *Total n* | *1544* |  |  | *124* |  |  | *153* |  |  | *1162* |  |  | *122* |  |
| **When last had a job** | |  |  |  |  |  |  |  |  |  |  |  |  |  |  |
|  | In paid work | 116 | 7.8 |  | 61 | 49.2 |  | 12 | 8.1 |  | 37 | 3.3 |  | 3 | 2.5 |
|  | 0-10 years | 728 | 49.2 |  | 48 | 38.7 |  | 92 | 62.2 |  | 538 | 48.5 |  | 66 | 55.0 |
|  | 11-20 years | *417* | 28.2 |  | *9* | 7.3 |  | *24* | 16.2 |  | *365* | 32.9 |  | *30* | 25.0 |
|  | >20 years | 120 | 8.1 |  | 2 | 1.6 |  | 6 | 4.1 |  | 103 | 9.3 |  | 8 | 6.7 |
|  | Never had a job | 99 | 6.7 |  | 4 | 3.2 |  | 14 | 9.5 |  | 66 | 6.0 |  | 13 | 10.8 |
|  | *Total n* | *1480* |  |  | *124* |  |  | *148* |  |  | *1109* |  |  | *120* |  |
| **Social class** |  |  |  |  |  |  |  |  |  |  |  |  |  |  |  |
|  | Management & professional | 271 | 18.1 |  | 34 | 27.4 |  | 15 | 10.1 |  | 202 | 18.0 |  | 23 | 18.9 |
|  | Intermediate | 158 | 10.5 |  | 15 | 12.1 |  | 15 | 10.1 |  | 121 | 10.8 |  | 12 | 9.8 |
|  | Small employers and own account | 104 | 6.9 |  | 14 | 11.3 |  | 6 | 4.1 |  | 69 | 6.2 |  | 3 | 2.5 |
|  | Lower supervisory & technical | 171 | 11.4 |  | 11 | 8.9 |  | 14 | 9.5 |  | 136 | 12.1 |  | 13 | 10.7 |
| Semi-routine, routine & never worked/LT | | 796 | 53.1 |  | 50 | 40.3 |  | 98 | 66.2 |  | 592 | 52.9 |  | 71 | 58.2 |
|  | *Total n* | *1500* |  |  | *124* |  |  | *148* |  |  | *1120* |  |  | *122* |  |
| **Housing tenure** | |  |  |  |  |  |  |  |  |  |  |  |  |  |  |
|  | owned | 286 | 18.5 |  | 9 | 7.3 |  | 11 | 7.2 |  | 245 | 21.1 |  | 19 | 15.6 |
|  | mortgage | 348 | 22.6 |  | 57 | 46.0 |  | 16 | 10.5 |  | 283 | 24.4 |  | 24 | 19.7 |
|  | rented | 907 | 58.8 |  | 56 | 45.2 |  | 126 | 82.4 |  | 632 | 54.4 |  | 79 | 64.8 |
|  | other | 2 | 0.1 |  | 2 | 1.6 |  | 0 | 0.0 |  | 1 | 0.1 |  | 0 | 0.0 |
|  | *Total n* | *1543* |  |  | *124* |  |  | *153* |  |  | *1161* |  |  | *122* |  |
| **Born in the UK** | |  |  |  |  |  |  |  |  |  |  |  |  |  |  |
|  | No | 143 | 9.3 |  | 12 | 9.7 |  | *19* | 12.4 |  | 104 | 8.9 |  | 9 | 7.4 |
|  | Yes | 1,401 | 90.7 |  | 112 | 90.3 |  | *134* | 87.6 |  | 1,059 | 91.1 |  | 113 | 92.6 |
|  | *Total n* | *1544* |  |  | *124* |  |  | *153* |  |  | *1163* |  |  | *122* |  |
|  |  |  |  |  |  |  |  |  |  |  |  |  |  |  |  |
| ***Appendix 13 cont. FURTHER DISABILITY COUNT / TYPE DATA*** | | | | | | |  |  |  |  |  |  |  |  |  |
|  |  |  |  |  |  |  |  |  |  |  |  |  |  |  |  |
| **Total disability count** | |  |  |  |  |  |  |  |  |  |  |  |  |  |  |
|  | 0 | 162 | 10.5 |  | 44 | 35.5 |  | 41 | 26.8 |  | 110 | 9.5 |  | 18 | 14.8 |
|  | 1 | 203 | 13.1 |  | 21 | 16.9 |  | 29 | 19.0 |  | 151 | 13.0 |  | 14 | 11.5 |
|  | 2 | 231 | 15.0 |  | 25 | 20.2 |  | 32 | 20.9 |  | 178 | 15.3 |  | 15 | 12.3 |
|  | 3 | 247 | 16.0 |  | 17 | 13.7 |  | 23 | 15.0 |  | 190 | 16.3 |  | 15 | 12.3 |
|  | 4 | 214 | 13.9 |  | 5 | 4.0 |  | 14 | 9.2 |  | 151 | 13.0 |  | 27 | 22.1 |
|  | 5 | 179 | 11.6 |  | 5 | 4.0 |  | 3 | 2.0 |  | 138 | 11.9 |  | 13 | 10.7 |
|  | 6 | 138 | 8.9 |  | 2 | 1.6 |  | 5 | 3.3 |  | 107 | 9.2 |  | 8 | 6.6 |
|  | 7 | 81 | 5.2 |  | 2 | 1.6 |  | 3 | 2.0 |  | 67 | 5.8 |  | 5 | 4.1 |
|  | 8 | 50 | 3.2 |  | 1 | 0.8 |  | 2 | 1.3 |  | 39 | 3.4 |  | 5 | 4.1 |
|  | 9 | 21 | 1.4 |  | 1 | 0.8 |  | 0 | 0.0 |  | 14 | 1.2 |  | 1 | 0.8 |
|  | 10 | 14 | 0.9 |  | 1 | 0.8 |  | 1 | 0.7 |  | 13 | 1.1 |  | 1 | 0.8 |
|  | 11 | 4 | 0.3 |  | 0 | 0.0 |  | 0 | 0.0 |  | 4 | 0.3 |  | 0 | 0.0 |
|  | 12 | 1 | 0.1 |  | 0 | 0.0 |  | 0 | 0.0 |  | 1 | 0.1 |  | 0 | 0.0 |
|  | *Total n* | *1545* |  |  | *124* |  |  | *153* |  |  | *1163* |  |  | *122* |  |
| **Disability type** | |  |  |  |  |  |  |  |  |  |  |  |  |  |  |
|  | Mobility | 967 | 63 |  | 37 | 30 |  | 61 | 40 |  | 761 | 65 |  | 73 | 60 |
|  | Lifting / carrying | 1008 | 65 |  | 46 | 37 |  | 70 | 46 |  | 778 | 67 |  | 81 | 66 |
|  | Manual dexterity | 496 | 32 |  | 21 | 17 |  | 16 | 10 |  | 385 | 33 |  | 36 | 30 |
|  | Continence | 302 | 20 |  | 11 | 9 |  | 13 | 8 |  | 235 | 20 |  | 22 | 18 |
|  | Hearing | 125 | 8 |  | 7 | 6 |  | 8 | 5 |  | 109 | 9 |  | 5 | 4 |
|  | Sight | 205 | 13 |  | 9 | 7 |  | 8 | 5 |  | 168 | 14 |  | 12 | 10 |
|  | Speech / communication | 147 | 10 |  | 2 | 2 |  | 9 | 6 |  | 110 | 9 |  | 11 | 9 |
|  | Memory / learning | 534 | 35 |  | 21 | 17 |  | 36 | 24 |  | 396 | 34 |  | 44 | 36 |
|  | Recognising physical danger | 125 | 8 |  | 6 | 5 |  | 7 | 5 |  | 92 | 8 |  | 4 | 3 |
|  | Coordination | 482 | 31 |  | 18 | 15 |  | 27 | 18 |  | 360 | 31 |  | 40 | 33 |
|  | Personal care | 437 | 28 |  | 14 | 11 |  | 20 | 13 |  | 324 | 28 |  | 38 | 31 |
|  | Other disability | 509 | 33 |  | 28 | 23 |  | 35 | 23 |  | 388 | 33 |  | 38 | 31 |
|  | *Sample total n* | *1545* |  |  | *124* |  |  | *153* |  |  | *1163* |  |  | *122* |  |

### Appendix 14: Summary of matching strategy, analysis 1 (pre-baseline sample only)

|  |  | *Off C-S* | *Mean* | *Median* | *Rubins' B** | *Rubins' R^* |
| --- | --- | --- | --- | --- | --- | --- |
|  |  |  |  |  |  |  |
| Mental health | Unmatched | 0 | 29.4 | 21.8 | 151.3 | 1.2 |
|  | Kernel 0.02 | 10 | 4.4 | 3.1 | 22.9 | 0.9 |
|  |  |  |  |  |  |  |
| Physical health | Unmatched | 0 | 29.4 | 22.0 | 151.0 | 1.2 |
|  | Kernel 0.02 | 9 | 3.5 | 2.0 | 20.1 | 0.9 |
|  |  |  |  |  |  |  |

### Appendix 15: Balance of covariates at t-1 between treatment and control groups before and after matching (pre-baseline sample only)

|  |  | Analysis 1 | | |
| --- | --- | --- | --- | --- |
|  |  | Treated | Control | %bias |
|  |  | (Kernel, bw 0.02) | | |
|  |  |  |  |  |
| Sex (Ref: male) | Before matching | 0.61 | 0.57 | 7.7 |
|  | After matching | 0.61 | 0.57 | 7.7 |
|  |  |  |  |  |
| Age in years | Before matching | 41.69 | 47.98 | -56.5 |
|  | After matching | 42.84 | 41.40 | 13.0 |
|  |  |  |  |  |
| Marital status (Ref: not married/civil p'ship) | Before matching | 0.38 | 0.42 | -7.6 |
|  | After matching | 0.39 | 0.40 | -1.7 |
|  |  |  |  |  |
| Region: N.England & Midlands | Before matching | 0.34 | 0.41 | -14.9 |
|  | After matching | 0.32 | 0.34 | -3.0 |
|  |  |  |  |  |
| Region: S.England & Wales | Before matching | 0.57 | 0.44 | 24.9 |
|  | After matching | 0.60 | 0.57 | 4.7 |
|  |  |  |  |  |
| Region: Scotland | Before matching | 0.07 | 0.10 | -10.1 |
|  | After matching | 0.06 | 0.07 | -2.4 |
|  |  |  |  |  |
| Region: N.Ireland | Before matching | 0.03 | 0.05 | -12.6 |
|  | After matching | 0.02 | 0.02 | -1.2 |
|  |  |  |  |  |
| Degree education (Ref: A-Level or lower) | Before matching | 0.30 | 0.20 | 21.9 |
|  | After matching | 0.27 | 0.21 | 14.2 |
|  |  |  |  |  |
| Long-standing illness or disability (Ref: Yes) | Before matching | 0.22 | 0.05 | 49.6 |
|  | After matching | 0.15 | 0.15 | -1.0 |
|  |  |  |  |  |
| General Health Questionnaire (0-36) | Before matching | 16.36 | 17.99 | -20.9 |
|  | After matching | 16.77 | 16.56 | 2.8 |
|  |  |  |  |  |
| Total disability count (0-12) | Before matching | 1.59 | 3.37 | -86.1 |
|  | After matching | 1.76 | 1.70 | 2.6 |
|  |  |  |  |  |
| Gross monthly income (£) | Before matching | 1241.50 | 1167.00 | 9.6 |
|  | After matching | 1279.00 | 1253.30 | 3.3 |
|  |  |  |  |  |
| Number of own children in household | Before matching | 0.57 | 0.36 | 23.1 |
|  | After matching | 0.56 | 0.66 | -10.0 |
|  |  |  |  |  |
| UK born (ref=no) | Before matching | 0.88 | 0.92 | -13.0 |
|  | After matching | 0.89 | 0.90 | -5.2 |
|  |  |  |  |  |
| House owner or mortgage (ref=no) | Before matching | 0.51 | 0.40 | 21.8 |
|  | After matching | 0.47 | 0.44 | 6.0 |
|  |  |  |  |  |
| Years since last job: in paid work at first wave | Before matching | 0.51 | 0.11 | 97.9 |
|  | After matching | 0.45 | 0.44 | 3.2 |
|  |  |  |  |  |
| Years since last job: <1 year to 10 years | Before matching | 0.35 | 0.47 | -24.9 |
|  | After matching | 0.39 | 0.40 | -3.1 |
|  |  |  |  |  |
| Years since last job: 11 to 20 years | Before matching | 0.07 | 0.28 | -59.2 |
|  | After matching | 0.08 | 0.08 | -0.5 |
|  |  |  |  |  |
| Years since last job: >20 years | Before matching | 0.01 | 0.07 | -29.6 |
|  | After matching | 0.02 | 0.02 | 0.5 |
|  |  |  |  |  |
| Years since last job: never had a job | Before matching | 0.05 | 0.06 | -4.2 |
|  | After matching | 0.06 | 0.06 | 1.3 |
|  |  |  |  |  |
| NS-SEC (1-5) | Before matching | 3.35 | 3.71 | -21.7 |
|  | After matching | 3.52 | 3.61 | -6 |

### Appendix 16: DiD-PSM for analysis 1 (pre-baseline sample only)

| **Analysis 1** |  | DiD with Propensity Score Matching (DiD-PSM) | | | | | |
| --- | --- | --- | --- | --- | --- | --- | --- |
|  |  |  |  |  |  |  |  |
|  |  | DiD-PSM | p-value | Lower CI | Upper CI | n | off CS |
|  |  |  |  |  |  |  |  |
| Mental health | | 6.26 | 0.001 | 2.45 | 10.07 | 798 | 10 |
| Physical health | | 1.35 | 0.381 | -1.67 | 4.37 | 801 | 9 |

*Note: DiD-PSM not carried out on subsample for analysis 2 & 3 because DID estimates non-significant due to smaller numbers – see appendix 12.*

### Web appendix References

Jenkinson, C., & Layte, R. (1997). Development and Testing of the UK SF-12. *Journal of Health Services Research, 2*(1), 14-18.

Jenkinson, C., Layte, R., Jenkinson, D., Lawrence, K., Petersen, S., Paice, C., et al. (1997). A shorter form health survey: can the SF-12 replicate results from the SF-36 in longitudinal studies? *Journal of public health medicine, 19*, 179-186.

Mcfall, S. (Ed.). (2013). *Understanding Society - UK Household Longitudinal Study: Wave 1-3, 2009-2012, User Manual.*: Colchester: University of Essex.

Rosenbaum, P. R., & Rubin, D. B. (1985). Constructing a Control Group Using Multivariate Matched Sampling Methods That Incorporate the Propensity Score. *The American Statistician, 39*(1), 33-38.

Rubin, D. (2001). Using Propensity Scores to Help Design Observational Studies: Application to the Tobacco Litigation. *Health Services and Outcomes Research Methodology, 2*(3-4), 169-188.

Ware, J. E., Kosinski, M., & Keller, S. D. (1996). A 12-Item Short-Form Health Survey: Construction of Scales and Preliminary Tests of Reliability and Validity. *Medical Care, 34*(3), 220-233.

Ware Jr, J. E. (2000). SF-36 health survey update. *Spine, 25*(24), 3130-3139.
